# Obstetrics, Gynecology and Pelvic Surgery

**Published:** 1896-05

**Authors:** William Warren Potter

**Affiliations:** Buffalo, N. Y., Examiner in obstetrics, New York State Medical Examining and Licensing Board


### OBSTETRICS, GYNECOLOGY AND PELVIC SURGERY.

CONDUCTED BY WILLIAM WARREN POTTER, M.D., Buffalo, N. Y.,
Examiner in obstetrics, New York State Medical Examining and Licensing Board.

### GONORRHEA IN THE FEMALE.

TAYLOR (*Charlotte Medical Journal*, quoted in *Medical Age*,) says that gonorrhea in women, as in men, consists of an exudative inflammation of the submucous connective tissue, and the genital organs of women are so extensive, complex and involuted, and so profusely supplied by blood-vessels, which frequently undergo normal engorgement, that it can readily be understood why the morbid process may show a tendency to become chronic and lurk and hide. There has been a proneness developed within the past ten years to refer, in a loose and unscientific manner, all female ailments to gonorrhea and attribute to many husbands, who in early days had been indiscreet, a gonorrheal infection of their wives, which produces serious consequences. The extreme and exaggerated views of Noeggerath, who claimed that 800 out of every 1,000 men living in large cities suffer from gonorrhea, which they have never recovered from, and who, on marrying, sooner or later infect their wives, have done much to perpetuate these ideas. There is a tendency nowadays to harp upon the longevity of the gonococcus, its phenix-like power of resuscitation and its relentless virulence. This idea, put forth by some syphilographers, has had undue weight with many gynecologists, who, under its influence are led to think that the gonococcus in the male and female never dies and is ever ready to produce pelvic mischief. I have seen many young women who have suffered from uterine and pelvic disease after marriage, whose trouble was induced by instrumental manipulation at the hands of energetic young men possessed of an ambition to be known as gynecologists. Minor surgery (Gynecology ?) is certainly the cause of a great many cases of uterine and pelvic disease. In estimating the importance of gonorrheal infection as a cause of female trouble, we must individualise, rather than generalise.

---

### STERILITY.

Grafe (*Centralbl. für Gynäk.*, No. 49, 1895, quoted in *Medical Record*,) gives the following causes of sterility :

1. Anomalies of the hymen or malformations of the genital tract. A very large vagina can also be a cause of sterility, as the sperma flows out immediately after coitus.
2. Vaginismus.
3. Excessive acid reaction of the vaginal mucus, which destroys the power of motion in the spermatozoa.
4. Narrow external or internal os, anteflexion, retroflexion, endometritis, gonorrhea, especially with involvement of the adnexa, neoplasms.
5. Constitutional diseases, as tuberculosis, syphilis, chlorosis and obesity.

---

### VAGINAL EXTIRPATION OF THE UTERUS AFTER LABOR.

Chrobak (*Centralbl. für. Gynäk.*, No. 21, 1895, quoted in *Kansas City Medical Record*,) is an advocate of this practice in cases of uncontrollable flooding where rupture is suspected, though he admits that the only two cases in which he acted on this principle died. In one case, a placenta-previa labor, violent hemorrhage set in during delivery and a laceration involving the cervix was detected. A tampon was applied, but rapidly became soaked, and a second was firmly packed into the uterine cavity. Hemorrhage continuing, Chrobak resolved to remove the uterus through the vagina, as that proceeding seemed to him simpler than hysterectomy from above. The operation was not very difficult. The uterus was nearly six inches long and as it proved difficult of extraction, a piece two finger-breadths wide was cut, like a slice made in an orange, out of the anterior wall. This permitted the organ to be easily withdrawn. There was very little hemorrhage during the removal of the uterus. Complete rupture of its wall was discovered. The laceration had apparently been enlarged when the second tampon was applied. The patient died. There are no details given of the second case. In one case the whole operation was done in four and the other in eight minutes. As far as the facility of extraction is concerned, Chrobak considers his experience encouraging.

———

FOR obstinate vomiting during early pregnancy, Dr. Baer (*Phil. Polyclinic*) recommends the following :

| | | |
|---|---|---|
| ℞ | Bismuth subnitrate | 2 drams. |
| | Saccharated pepsin | 1 dram. |
| | Sodium bicarbonate | ½ dram. |
| | Sugar of milk | 1 dram. |

Mix and make twelve powders. One every three hours.

In addition to the above the following prescription will be found to be most pleasing and effective :

| | | |
|---|---|---|
| ℞ | Diluted nitrohydrochloric acid | 1½ fl. drams. |
| | Spirit of lemon | 1 dram. |
| | Simple syrup | 2 ounces. |

Mix. Give one teaspoonful in a wineglass of ice water three times a day.

———

STATISTICS OF VAGINAL HYSTERECTOMY FOR UTERINE CARCINOMA.

SCHMID (*Centralblatt für Gynäkologie*, No. 43, 1895 ; *Univ. Med. Mag.*,) reports the results of forty-two cases where vaginal hysterectomy was performed for carcinoma. Thirty were carcinoma of the cervix, seven carcinoma of the corpus uteri, and two a carcinomatous degeneration of myoma. Up to 1892 he had operated upon thirty-four cases, with the following results : Seven died through operation, seven are free from return of the disease, twelve have a return, and eight failed to answer his communication.

### DIGITAL EXPLORATION IN MIDWIFERY.

CROUZAT, (*Revue Obstetrique Internationale; Univ. Med. Mag.*,) October 21, 1895, disagrees with certain German obstetricians who oppose digital exploration in normal labor and rely upon abdominal palpation. The diagnosis of normality may demand the introduction of the finger into the vagina. Crouzat's principles simplify digital exploration and guard against its dangers. Vaginal examination, he thinks, should be made as rarely as possible. One exploration at the beginning of labor and another immediately after the rupture of the membranes are usually sufficient. It is his practice to make the external parts antiseptic; the hands and forearms are then washed and brushed thoroughly. The nails must receive special attention. The washing is afterward repeated in a 1-1000 bichloride of mercury solution. Great care in the introduction of the forefinger is strongly advocated. This should be dipped in sublimated vaseline and guarded by the thumb and other fingers, whilst the hand is passed under the clothes and near the patient's thighs. On reaching the perineum the labia are parted by the thumb and middle finger. The forefinger is lastly introduced into the vagina without having touched any part of the patient or her clothes since the time it was made aseptic.

---

### THE MANAGEMENT OF MISCARRIAGE.

BARTON (*Med. Bulletin*) says two methods present themselves, one being what is called conservative and the other the active plan of treatment. He discussed the two methods and gave the reasons for his conviction as to which is the better. The two plans, briefly stated, are these: The one is to allow the placenta to remain and come away as best it may and the other is to forcibly remove the afterbirth, completely emptying the uterus of all the secundines. In the first method of treatment a foreign body is left in the uterus which is liable to become septic at any time, and when it does a woman's life is put in jeopardy, or if she escapes with her life, her health is almost sure to be permanently injured. Not only is she in danger of sepsis, but as long as the afterbirth is retained she is in danger of hemorrhage, which may be sufficient to terminate the woman's existence. In France this method is followed to a considerable extent. Tarnier is one who advocates it strongly, claiming that the womb should be allowed time to expel its contents. He points out hospital statistics in which he saw forty-six cases of retained placenta after abortion and only one death and that from pneumonia ; but the death-rate at a hospital in Florence, in which Tarnier's plan of treatment seems to have been carried out, shows a mortality of 6 per cent. Tarnier and Cazeau report a case as follows: "During the first five days the patient did very well, but on the sixth, and at three o'clock in the afternoon, a violent chill came on, which lasted an hour. This unfortunate lady died on the tenth day." All the advocates of this so-called conservative plan of treatment agree that in case there is any indication of sepsis, or even before that, when the lochia becomes offensive, the uterus should be emptied. The only dangers he can conceive of in the active plan of treatment are the introduction of septic material by the physician, which is inexcusable, and the danger of perforation of the uterus by the use of the curette. He is inclined to believe that in many of these cases the curetting did not immediately follow the abortion, but was done to remove a decomposing placenta in a softened uterus. Granting that there have been accidents, the percentage cf mortality from this cause is small as compared with the percentage of mortality in retained placenta. In the latter plan of treatment it is not only the death-rate but the injury to health that should count against it. We may have, as a result of a retained afterbirth, septic endometritis and salpingitis, with pyosalpinx ; we may have uterine phlebitis ; we may have septic phlebitis of one or both legs; we may have septic peritonitis, or septic pneumonia, or septic pleurisy and many other conditions to which the absorption of septic material gives rise. This, together with the danger of a fatal hemorrhage, which is constantly present in a retained placenta, is, when set over against the fact that a few women have lost their lives by the uterus being perforated with the curette in the active or radical method of treatment, it seems sufficient to decide the question in favor of the active method.

---

### HYDROCELE OF THE LABIUM MAJUS.

Edwards (*American Journal Obstetrics*) says : A prolongation of peritoneum may reach below the mons veneris through the inguinal ring, covering the round ligament. This peritoneal investment may become adherent above the ring, and a transudation of serum occur into the cavity formed. This condition is then known as hydrocele of the labium majus. He gives several varieties : 1. That in which there exists a patulous canal of Nuck. The fluid is excreted from the peritoneal surfaces covering the ligament and is free to return within the general peritoneal cavity. 2. The sac may be entirely cut off from the abdominal cavity and dropsy occur in this closed sac. 3. The cellular tissue of the labium majus consists of two layers, which are prolongations of the superficial abdominal fascia. These two layers are considered the analogue of the dartos tunic and between them a serous tumor may form. This is considered by some to be true hydrocele in woman. 4. The substance of the round ligament itself may be the site of a cyst. The gubernaculum of Hunter in the fetus betus becomes the round ligament in the female. This fetal structure is at first hollow and there may be a persistence of this fetal condition which allows the formation of a cyst. Eisenhart has collated forty-eight cases of hydrocele in the female, and finds that twenty-nine were upon the right side and nineteen upon the left. He considers traumatism and congenital defect to be the most frequent causes. Smith believes that the disease is not so rare as is stated ; during a period of four years, he says, five cases have been operated upon in the Tottenham hospital. The treatment of hydrocele feminina is operative. Expose the cyst by a linear incision, ligate the neck and enucleate. The wound is to be closed by superimposed layers, as in the closure of hernia. Simple puncture of the hydrocele is of little avail.

---

### ASAFETIDA IN OBSTETRICS.

WARMAN (*British Medical Journal*, quoted in *Md. Med. Jour.*,) finds that this drug is a most valuable therapeutic agent in midwifery. It is a direct sedative to the pregnant uterus and exercises no evil influence over the general system. It is of particular value when abortion is imminent, as it controls uterine irritability. On the other hand, it is of no use as a prophylactic agent in such cases and must not be relied upon when the abortion has proceeded so far as to require manual interference. In habitual constipation and in nervous conditions during pregnancy it is highly beneficial.

---

COLD BATHING DURING MENSTRUATION.—Cold bathing during menstruation is a beneficial measure, provided women accustom themselves to it by bathing every day for at least eight days before the period. Houzel holds that cold salt-water baths facilitate the menstrual flow, increase the duration of genital life and increase fecundity.—*Depasse, Lancet-Clinic.*

